# Development of a Novel Hanging Drop Platform for Engineering Controllable 3D Microenvironments

**DOI:** 10.3389/fcell.2020.00327

**Published:** 2020-05-07

**Authors:** Chin-Yi Cho, Tzu-Hsiang Chiang, Li-Hung Hsieh, Wen-Yu Yang, Hsiang-Hao Hsu, Chih-Kuang Yeh, Chieh-Cheng Huang, Jen-Huang Huang

**Affiliations:** ^1^Department of Chemical Engineering, National Tsing Hua University, Hsinchu, Taiwan; ^2^Institute of Biomedical Engineering, National Tsing Hua University, Hsinchu, Taiwan; ^3^Department of Biomedical Engineering and Environmental Sciences, National Tsing Hua University, Hsinchu, Taiwan; ^4^Department of Nephrology, Kidney Research Center, Linkou Chang Gung Memorial Hospital, Taoyuan, Taiwan; ^5^College of Medicine, School of Medicine, Chang Gung University, Taoyuan, Taiwan

**Keywords:** hanging drop, array, microtissue, glomerulus, podocyte

## Abstract

Conventional biomedical research is mostly performed by utilizing a two-dimensional monolayer culture, which fails to recapitulate the three-dimensional (3D) organization and microenvironment of native tissues. To overcome this limitation, several methods are developed to fabricate microtissues with the desired 3D microenvironment. However, they tend to be time-consuming, labor-intensive, or costly, thus hindering the application of 3D microtissues as models in a wide variety of research fields. In the present study, we have developed a pressure-assisted network for droplet accumulation (PANDA) system, an easy-to-use chip that comprises a multichannel fluidic system and a hanging drop cell culture module for uniform 3D microtissue formation. This system can control the desired artificial niches for modulating the fate of the stem cells to form the different sizes of microtissue by adjusting the seeding density. Furthermore, a large number of highly consistent 3D glomerulus-like heterogeneous microtissues that are composed of kidney glomerular podocytes and mesenchymal stem cells have been formed successfully. These data suggest that the developed PANDA system can be employed as a rapid and economical platform to fabricate microtissues with tunable 3D microenvironment and cellular heterogeneity, thus can be employed as tissue-mimicking models in various biomedical research.

## Introduction

Native tissues are comprised of multiple micro-scaled subunits that are characterized by distinct cellular heterogeneity, well-defined three-dimensional (3D) architectures, and tissue-specific niches (Liu et al., 2014; Todhunter et al., 2015; Kuo et al., 2017). Until now, the majority of our knowledge of cell behaviors and how they are modulated are learned through observations *in vitro* (Zakrzewski et al., 2019). However, current biomedical research is mostly carried out using two-dimensional (2D) cultured cells, which fail to faithfully recreate the complexity of the 3D microenvironment in living tissues (Ranga et al., 2014). As a result, it is not surprising that the observed cellular behavior and responses in such settings cannot appropriately reflect those *in vivo*. This dramatically hinders the progress in several research domains, including cell biology, drug discovery/development, disease modeling, and regenerative medicine (Fatehullah et al., 2016).

In recent years, techniques to engineer the 3D microenvironment *in vitro* have attracted considerable attention. This is because the cells cultivated in 3D configuration are exposed to the milieu that is more similar to *in vivo* tissues compared to that grown in conventional 2D configuration (Oliveira et al., 2018). More specifically, 3D-cultured cells exhibit several *in vivo*-like characteristics in terms of the morphology, behavior, metabolism, cellular heterogeneity, and cell-to-cell or cell-to-matrix interactions, which are all diminished in the 2D-cultivated cells (Kuo et al., 2017; Oliveira et al., 2018; Kim et al., 2019). Therefore, cells cultured in 3D configuration and engineered to micro-scaled tissues represent a better *in vitro* model that recapitulates the physiological conditions of native tissues and has emerged as powerful tools for biomedical applications.

Currently, several approaches (hanging drop, centrifugal pellet, spinner flask, patterned surface, and magnetic or acoustic levitation of cells) and materials (non-adhesive substrates, superhydrophobic/superhydrophilic surfaces, and porous 3D scaffolds) have been developed as culture platforms for 3D microtissue fabrication (Lin and Chang, 2008; Achilli et al., 2012; Cui et al., 2017; Yu et al., 2020). Despite these being proven to be useful for generating 3D microtissues, each of these methods may have some limitations that hinder their widespread application in various biomedical domains. The conventional hanging drop technique is labor-intensive and requires a second manipulation for transferring the formed microtissue into a different culture vessel for the subsequent assays (Fang and Eglen, 2017). Additionally, the formed microtissues may exhibit various sizes and morphologies (Ouyang et al., 2015). However, even though microtissues with a relatively uniform geometry can be obtained by using external forces to aggregate cells, the subtle cellular responses toward the applied physical forces can affect the biochemical and physiological functions of the cells, thus altering their behavior or even viability (Skiles et al., 2013). Using novel materials as substrates for 3D microtissue cultivation can be highly efficient but extremely expensive, thus not being cost-effective for mass production (Liao et al., 2019). Therefore, developing a 3D microtissue culture platform that has a high level of reproducibility and consistency along with it being economical and easy to operate, would be highly favored to advance the laboratory proof-of-concepts to develop clinically usable tools.

To address the above-mentioned drawbacks of the existing approaches, we present a pressure-assisted network for droplet accumulation (PANDA) system to form a consistent and uniform hanging drop array for fast and massive production of native-like 3D microtissues, enabling the easy establishment of controllable and adjustable 3D microenvironments *in vitro* for further applications. As a proof of concept, the PANDA system was employed for the fabrication and study of the kidney glomerular microtissues that were composed of podocytes and mesenchymal stem cells (MSCs). The podocytes were post-mitotic and specialized glomerular epithelial cells that contribute to renal filtration barrier (May et al., 2014; Hale et al., 2018), while the MSCs could differentiate into mesangial cells that are crucial pericytes in glomeruli (Imasawa et al., 2001; Wong et al., 2014). Drug-induced nephrotoxicity, which leads to podocyte loss, glomerular scarring, and thus, acute or chronic kidney injury, remains a challenging issue in clinical settings and preclinical drug development (Kandasamy et al., 2015; Musah et al., 2017). Using the developed PANDA chip, a large number of kidney microtissues with a uniform composition, which is an essential criterion for the reproducibility of high-throughput screening, could be generated easily. Therefore, the PANDA chip developed in this study holds significant potential to serve as a rapid and economical platform for the growth of highly consistent microtissues with precisely controlled and tunable 3D microenvironments for use in cell biology research, drug screening, therapeutic effect prediction, and engineering implantable tissues.

## Principle

**FIGURE 1:**
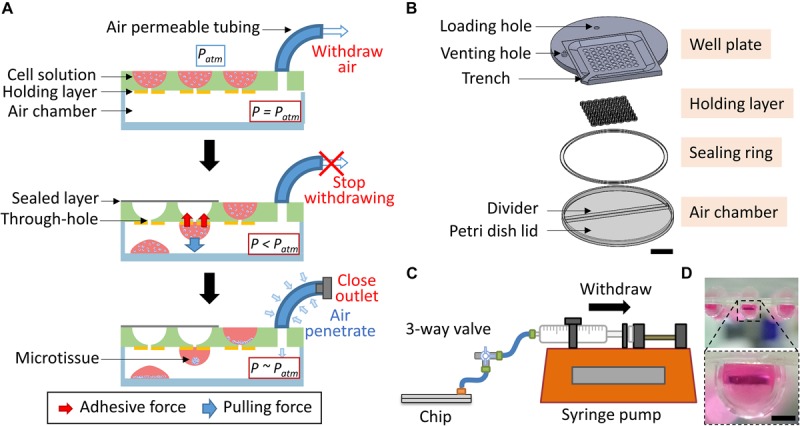
The PANDA system. **(A)** Schematic of the PANDA principle. The internal air of the system is withdrawn from the air chamber, leading to the pressure of the air chamber (*P*) being less than the atmosphere pressure (*P*_atm_). The pressure difference allows the cell solution located on the well plate to enter into the air chamber. The cohesive force causes the cell solution to form a droplet while the adhesive force from the holding layer prevents the droplet from dripping that eventually forms the hanging drop. The PANDA system can be placed in the incubator and each hanging drop can allow the cells to spontaneously form the spheroid (or microtissue). The air can gradually penetrate through a gas permeable tubing to balance the internal pressure and the surrounding pressure. **(B)** The exploded view of the PANDA system. Scale bar represents 2 cm. **(C)** The schematic of the PANDA system connected to a syringe pump. **(D)** The hanging drops formed after using the PANDA system. Scale bar represents 1 mm.

The major goal of this study is to form consistent hanging drops spontaneously by using a cost-effective approach for the generation of 3D microtissues in a controllable manner. To achieve this goal, a mechanism based on the pressure difference between the surrounding environment and the system was applied to spontaneously pull the cell suspension toward the internal chamber to form the hanging drops (Figure 1A). The pressure difference was generated by withdrawing the air from the internal air chamber through air permeable tubing. Initially, the pressure inside the air chamber (*P*) was the same as the atmosphere pressure (*P*_atm_). Once the air was withdrawn from the air chamber, the *P* was less than *P*_atm_, which led to the cell suspension located on the well plate overcoming the capillary force and entering the internal chamber through the through-hole. The penetrated cell suspension could spontaneously form a spherical droplet due to the cohesive force and adhered to the wall because of the surface tension. The pulling force combined with the pressure-driven force and gravity force was required to be less than the adhesive force to prevent the dripping of the droplet. On the contrary, an insufficient pulling force would not be able to overcome the capillary force to form the hanging drop which served as a microenvironment for cell growth. Therefore, a high adhesive force generated from the holding layer was essential to balance the pulling force and to maintain the hanging status of the droplet. This could be achieved by manipulating the hydrophilic properties between the well plate and the holding layer. In general, highly hydrophilic materials can hold the droplet without dripping but may lead to more liquid residue remaining on the wells or flatten the edge of the hanging drop. Once the droplet was formed, the withdrawal of air was stopped, and the outlet of the system was closed to finish the forming procedure. The PANDA system could be placed on the incubator, therefore, the *P* could gradually equal *P*_atm_ due to more air entering into the internal chamber through a gas permeable tubing connected to the PANDA system. This ensured that the whole system provided a normal culturing condition for the growth of the cells. Compared to the conventional hanging drop method, the PANDA system contributed to the development of an efficient method to culture the cells using the hanging drop method.

## Materials and Methods

### Design and Fabrication of the PANDA System

To balance the pulling force and the adhesive force, an air-sealed chamber, and a material with a slightly high hydrophilic surface were two critical aspects of the PANDA system. The PANDA system consisted of four major parts: the well plate, holding layer, sealing ring, and air chamber (Figure 1B). Each part was designed using a 3D CAD design software (SolidWorks 2019, Dassault Systèmes, United States) and followed the same design logic similar to the previous study (Lin et al., 2019). The shell-shaped well plate contained a circular region with a diameter of 85 mm and a square with each side 70 mm. The thickness of the well plate was set to 4.5 mm and the diameter of each well was 4.6 mm, thus allowing to hold more medium for the long-term growth of cell culture. For the region generating the hanging drop, the well plate was scooped in a 45.4 mm square area with 1.5 mm depth to gather extra liquid when pouring the medium on the plate. In the square groove, 49 wells with a diameter of 4.6 mm were designed to accommodate cell suspension. At the bottom of the well, a penetrating channel of 1.2 mm diameter was designed to keep the cell suspension in the well but allow it to penetrate when the pressure difference was generated. Furthermore, a trench was designed to connect to the square-shaped groove at the edge of the plate to discharge the excess cell suspension. The well plate was constructed by engraving a 4.5 mm thick polycarbonate (PC) sheet (Formosa Idemitsu Petrochemical Corporation, Taiwan) using a CNC milling machine (Roland MDX-40A Benchtop CNC Mill, United States). A venting hole of 4 mm diameter was engraved on the top of the well plate so that the tubing adaptor (BDMR210-9, Nordson MEDICAL, United States) could be installed and fixed with epoxy glue to facilitate the assembling of the chip.

The holding layer was designed to have a connecting ring structure corresponding to the position of the wells on the well pate. Once the holding layer was assembled with the well plate, only the hydrophilic rings could hold the droplets while other regions prevented the spread of the formed droplets (Hsiao et al., 2012). To investigate the adhesive force of the holding layer, we focused on the geometry and hydrophilicity of the ring. We chose polyethylene terephthalate (PET, Formosa Idemitsu Petrochemical Corporation, Taiwan) as the material for the holding layer because it had higher hydrophilicity (contact angle = 72.5°) than PC (contact angle = 82°). Therefore, when the droplets formed under the chip, the hydrophilic property of PET stabilized the droplets, preventing them from dripping. The holding layer was generated by alternatively assembling the upper part (named as U) and the lower part (named as L) to study the influence of the microstructure. The ring structure of the upper part had 4.6 mm outer diameter and 1.2 mm inner diameter while the ring structure of the lower part had 4.6 mm outer diameter and 2.6 mm inner diameter (Supplementary Figure S1). Both parts were designed using the Solid Edge 2D software package (ST9, Siemens, United States) and fabricated using a layer-by-layer stacking technique on the PET sheets (Ni et al., 2019). The PET sheets were pre-laminated with double-sided adhesive tape (9122, 3M Company, United States) and machined using a laser cutting machine (PLS6.75, Universal Laser System, United States) so that the holding layer could be assembled with the well plate to generate specialized geometries.

A 90 mm diameter petri dish lid (66-1501, Biologix, China) was used as the air chamber of the PANDA system. A customized divider (76 mm length) was cut from the PC sheet by using the CNC milling machine and inserted into the petri dish cap to create the air chamber and phosphate-buffered saline (PBS) loading reservoir. The sealing ring was cut from the 0.1 mm thick PET sheet pre-laminated with the double-sided adhesive tape by using the laser cutter to obtain the ring structure with 88 mm inner diameter and 96 mm outer diameter (Supplementary Figure S2A). The PANDA chip was prepared by placing the well plate on the air chamber and sealing with the sealing ring to avoid leakage during the air withdrawal process. The sealing sheet of size 46 × 46 mm for covering the well plate, was cut from the PET sheet (0.1 mm thickness) pre-laminated with the adhesive tape using the laser cutter (Supplementary Figure S2B). All the devices and connecting components used for the preparation of the cell culture were sterilized following the standard autoclave procedure.

### Setup of the PANDA System

As shown in Figure 1C, the PANDA system was set up by connecting the well plate and a syringe (10 mL, Becton Dickinson, United States) installed in a syringe pump (NE-4000, New Era Pump Systems, United States) through a silicone tubing (1/16 inch inner diameter, EW-95802-02, Cole-Parmer, United States) to generate a constant and steady withdrawal of air from the PANDA chip. The connection between the silicone tubing and syringe was made using a female Luer fitting (FTLL210-9, Nordson MEDICAL, United States). A Luer 3-way valve (Guangzhou JU Plastic Fitting Technology, China) was installed between the PANDA chip and syringe pump, allowing the disconnection of the PANDA chip from the syringe pump after the formation of the hanging drops.

### Analysis of Internal Pressure

The internal pressure of the chamber was analyzed by using a pressure sensor system. Briefly, another 3-way valve (serving as a venting valve) was connected after the original 3-way valve (serving as a connection valve) that had been connected to the PANDA chip (Supplementary Figure S3). A differential pressure transmitter (984, Beck Sensors, Germany) was connected to the venting valve to monitor the internal pressure within the system in real-time. All the connections were made using Luer lock adaptors and silicone tubing. The real-time data of the internal pressure was acquired by a portable data acquisition module (USB-4718, Advantech, Taiwan), which could transfer and record the data in the computer for analysis later.

### Measurement of Success Rate

The successful and consistent formation of the hanging drops is critical for establishing a stable microenvironment to culture the cells. Failures in the formation of hanging drops such as dripping, flatting, and even non-response may influence the other drops or reduce the testing samples for further experiments. To verify the various assembling and operating conditions of the PANDA system, the success rate of hanging drop formation was estimated by using Equation 1 as follows.

Successrate(%)

(1) $$=\frac{\mathrm{Number}\,\mathrm{of}\,\mathrm{drops}\,\mathrm{attached}\,\mathrm{on}\,\mathrm{the}\,\mathrm{plate}}{\mathrm{Number}\,\mathrm{of}\,\mathrm{the}\,\mathrm{wells}}\times 100$$

### Cell Culture

Conditionally immortalized mouse podocytes, derived by Endlich’s group, which were transfected with a temperature-sensitive T antigen and green fluorescent protein (GFP), were used in this study (Schiwek et al., 2004; Chen et al., 2018). The podocytes were maintained at 33 °C in RPMI 1640 medium (Thermo Fisher Scientific, United States) and supplemented with 10% fetal bovine serum (FBS; GE Healthcare Bio-Sciences, United States), 10 U/mL mouse interferon (IFN)-γ (PeproTech, United States) that drove the T-antigen application (Chen et al., 2018). To deactivate the T antigen and promote podocyte differentiation, cells were incubated at 37°C, and their culture medium was changed to that without IFN-γ (Chen et al., 2018).

Human umbilical cord blood-derived MSCs that were transfected with red fluorescence protein (RFP) were obtained from the Bioresource Collection and Research Center, Food Industry Research and Development Institute, Hsinchu, Taiwan (Hung et al., 2010). The MSCs were cultured in α-minimum essential medium (Thermo Fisher Scientific, United States) supplemented with 20% FBS and 4 ng/mL basic fibroblast growth factor (PeproTech, United States).

### Fabrication and Characterization of the 3D Microtissues Using PANDA System

For the formation of 3D kidney microtissues, the confluent podocytes and MSCs were harvested from culture dishes using 0.05% trypsin and mixed with 1:1 ratio of cell density. The cell mixture was then transferred into a PANDA chip followed by the hanging drop formation procedure to culture the cells. The grown microtissues were observed daily under a fluorescence microscope (Olympus, Japan) to measure the diameter of the 3D microtissues. Alternatively, the 3D microtissues were collected and processed for immunofluorescence staining. Samples were fixed in 4% paraformaldehyde, permeabilized with 0.1% Triton X-100, and incubated with a blocking buffer for 1 h followed by primary antibody against P-cadherin (1:200 dilution; Abcam, United States) staining at 4°C overnight. Alexa Fluor 633-conjugated secondary antibody (1: 200 dilution; Thermo Fisher Scientific, United States) was used to detect the primary antibody. The nuclei were visualized by counterstaining with 4’,6-diamidino-2-phenylindole (DAPI; 1 μg/mL; Thermo Fisher Scientific, United States). The mounted samples were imaged under a laser scanning confocal microscope (LSM 780, Carl Zeiss, Germany). Additionally, a Live/Dead Viability/Cytotoxicity Kit (Thermo Fisher Scientific, United States) was used to evaluate the viability of the cells grown within 3D microtissue according to the manufacturer’s instruction. In order to prevent crosstalk of fluorescence, the podocytes and MSCs that were not expressing fluorescence proteins were employed for the live/dead assay.

### Statistical Analysis

Data were obtained from three independent experiments and analyzed through one-way ANOVA with Bonferroni correction using GraphPad Prism (Version 8.3, GraphPad Software, United States). The data are expressed as mean ± standard deviation. Results were considered significant when ^∗^*P* < 0.05 and ^****^P < 0.001.

## Results and Discussion

### Manipulation of PANDA System

**FIGURE 2:**
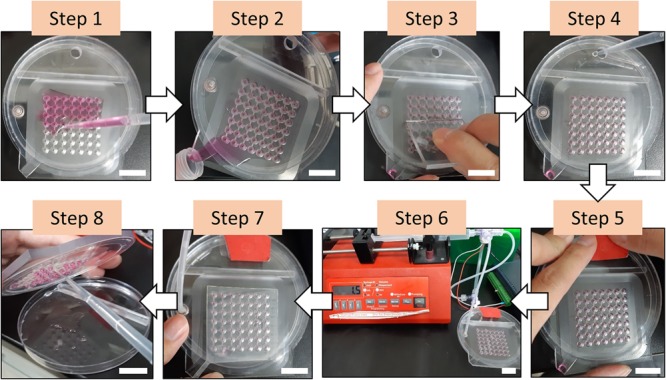
The procedure of forming the hanging drops using the PANDA system. **Step 1**: Add the cell suspension on the well plate. **Step 2**: Pour the excess cell suspension into a centrifuge tube. **Step 3**: Use a scraper to remove the residue. **Step 4**: Fill the reservoir with PBS through the loading hole. **Step 5**: Seal the loading hole. **Step 6**: Connect with the syringe pump to initiate the withdrawal of air and wait until all hanging drops are formed. **Step 7**: Seal the wells using the sealing sheet. The PANDA system is now ready for cell culture. **Step 8**: After cell culture, disassemble the chip and collect the hanging drops using a pipette for further experiments. All scale bar represent 2 cm.

The hanging drop technique has been widely employed to form microtissues from primary cells or stem cells due to its simplicity and ease of operation without using specialized equipment (Laschke and Menger, 2017; Shri et al., 2017). Furthermore, this approach can achieve the desired uniform size of the microtissues, which is a critical factor to reproduce the experimental results for drug screening compared to other approaches (Mehta et al., 2012). However, the extremely labor-intensive operating procedure limits the development of this technique for high throughput experiments (Amaral et al., 2017). Manually loading the cell suspension on the hanging drop plate has been verified with high size variation of the produced microtissues, although the variation is still relatively low (SD ∼10%). Although this tedious operating procedure that requires careful handling has been overcome by introducing robotic liquid handling equipment (Drewitz et al., 2011; Tung et al., 2011), this complicated and costly instrument is essential to obtain consistent results. Here, we have introduced the PANDA system to produce the hanging drop array spontaneously and consistently using only a commercially available syringe pump. The operating procedure for the PANDA system is straightforward. There is total of eight major steps to form the hanging drops for the growth of microtissues (Figure 2). Step 1: Add the cell suspension on the well plate by using a pipette. Similar to other approaches for microtissue formation, the cell concentration that controls the size of the microtissue can be determined at this step. Step 2: Pour the excess cell suspension into a centrifuge tube. The collected cell suspension can be used in the next chip. Step 3: Use a scraper to remove the residue. Notably, this step should be repeated until the level of the solution in the well is uniform and can ensure that each well retains the same amount of cell suspension to obtain the uniform size of the hanging drops. Step 4: Fill the reservoir with PBS through the loading hole to retain moisture inside the air chamber during the cell culture. Step 5: Seal the loading hole to enclose the whole system so that air can only be withdrawn from the venting hole. Step 6: Connect with the syringe pump to initiate the withdrawal of air. The hanging drops are formed individually with time and can be visualized from the top view (Supplementary Video S1). When the last hanging drop is formed, stop the syringe pump. Over-withdrawal of air triggers the dripping of the hanging drops. Step 7: Seal the wells using the sealing sheet to prevent the hanging drops from evaporating, so that the air chamber can maintain lower pressure and prevent the backflow of the cell suspension. After switching off the 3-way valve, the PANDA chip can be disconnected and placed in the cell culture incubator until the microtissues are formed. Step 8: After removal from the cell culture, disassemble the chip and collect the hanging drops using a pipette. The hanging drops can be collected individually or collectively for further experiments. The size of the hanging drops from the 49-wells PANDA chip was measured to be 13.75 ± 0.24 μL, demonstrating the uniform formation of hanging drops using the procedure developed in this study.

### Adhesion Capability of Holding Layer

**FIGURE 3:**
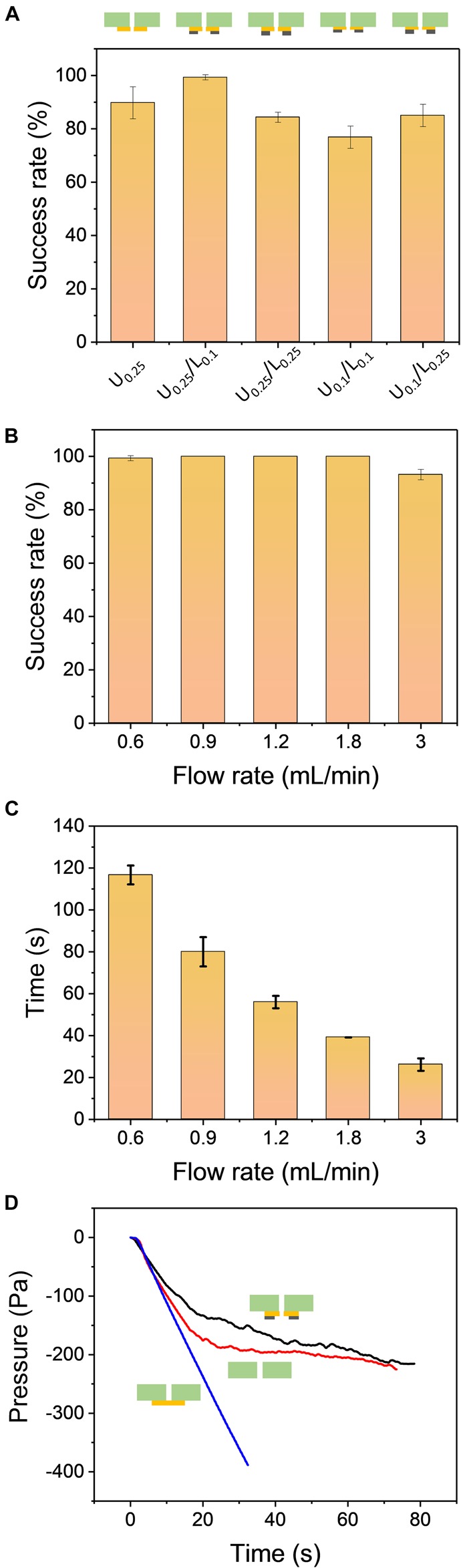
**(A)** The comparison of the hanging drop formation success rate using five different combinations of the holding layers: U_0.25_, U_0.25_/L_0.1_, U_0.25_/L_0.25_, U_0.1_/L_0.1_, U_0.1_/L_0.25_. The flow rate of air withdrawal was set to 0.9 mL/min. **(B)** The comparison of the success rate for different flow rates. The design of the holding layer was U_0.25_/L_0.1_. **(C)** The processing time of hanging drop formation when using various flow rates. All results were repeated three times (*N* = 3). **(D)** The internal pressure of the air chamber during operation of the PANDA system at different holding conditions: sealed holding layer (blue line), without holding layer (red line), and U_0.25_/L_0.1_ design of the holding layer (black line). The flow rate of air withdrawal was set to 0.9 mL/min.

The formation of the hanging drops is based on the air pressure-driven force to pull the cell suspension entering the through-hole, while the adhesive force from the holding layer holds the hanging drops and prevents dripping. There are two major factors to maintain the high adhesive force of the holding layer: hydrophilicity and surface geometry. A high hydrophilic surface can be achieved by coating the surface with hydrophilic materials such as polydopamine (Lee et al., 2007; Kang et al., 2012), but the additional coating step and cost limit the use in further applications and for mass production. Most importantly, these materials may interfere with the growth of the cells during cell culture. High hydrophilicity of the holding layer can prevent the hanging drop from dripping but it can also trigger the spreading of the liquid on the surface, leading to failure to form a spherical droplet. The micro-topographical features of the surface have been reported to stabilize the droplet arrays without spreading on the surface and spheroids have been successfully formed inside the hanging drops (Hsiao et al., 2012). To enhance the adhesion capability of the holding layer, we generated five different microstructures using a more hydrophilic material such as PET compared to the material of the well plate (PC). Both the upper and lower parts of the holding layer had a thickness of 0.1 mm and 0.25 mm so that the combinations of the holding structure were U_0.25_, U_0.25_/L_0.1_, U_0.25_/L_0.25_, U_0.1_/L_0.1_, and U_0.1_/L_0.25_. Based on the results from Figure 3A, the holding layer design with U_0.25_/L_0.1_ had an almost 100% success rate while the other designs had success rates ranging from 78 to 90%. The upper part with 0.25 mm thickness resulted in a high success rate compared to the upper part with 0.1 mm thickness, suggesting that the upper part played a major role in contributing high adhesive force even without the lower part. Therefore, the holding layer with U_0.25_/L_0.1_ microstructure was chosen in the following experiment.

### Influence of Flow Rate

Although providing a constant withdrawal of air is sufficient to accomplish the formation of hanging drops, the flow rate controlled by the syringe pump can determine the total operation time in the whole process. To investigate the influence of the flow rate, the success rates at five different flow rates were measured. When the PANDA system was operated to form the hanging drop array, only the flow rate at 3 mL/min had a success rate of 93% while the other flow rates had success rates up to 100% (Figure 3B). This flow rate generated a sudden change of air pressure that led to a high pressure-driven force to pull the liquid. However, it also caused the liquid to have insufficient time to spread on the hydrophilic surface of the holding layer. Therefore, we observed that 2–3 out of 49 wells were not able to attach firmly on the chip. We observed that one well failed to hold the hanging drop at the flow rate equal to 0.6 mL/min. This is because the pressure-driven force was slowly generated in the air chamber, leading to the unstable formation of the hanging drops. Furthermore, we investigated the processing time to completely generate the 49 hanging drop arrays at different flow rates and studied whether the operating process was efficient in forming the hanging drops compared to the conventional pipette based approach. The operating time was defined as the interval from the start of the syringe pump to the formation of the last droplet. Figure 3C demonstrates that at a low flow rate of 0.6 mL/min, the processing time was approximately 117 s to form 49 hanging drop arrays, while at a high flow rate up to 3 mL/min it required only 26 s to complete the formation process. We tested the processing time to manually load 48 wells of 20 μL liquid in a 96-well plate using an 8-channel pipette. It required 60–90 s of processing time depending on the number of persons. Thus, the PANDA system not only generated a uniform hanging drop array, but also increased the operational efficiency and reduced the human errors in manual operation.

### Internal Pressure of the PANDA System

The one-by-one formation process of hanging drops can be visualized from the top of the chip with naked eyes. It can also be used to determine the end of the operation process by observing the formation of the last hanging drop. This approach is sufficient when a few hanging drops are to be determined but may become tedious when a large number of hanging drops are formed. To decide when to stop the syringe pump, it is more effective to analyze the internal pressure of the air chamber in real-time. Figure 3D demonstrates that the internal pressure of the air chamber changed with time during the air withdrawal process. Three different conditions were compared in the PANDA system: sealed holding layer, without holding layer, and the holding layer with U_0.25_/L_0.1_ design. Initially, the internal pressure at three different conditions all decreased when the syringe pump began to withdraw the air at a constant flow rate. The pressure linearly decreased to −350 Pa at 30 s for the condition with the sealed holding layer. It is reasonable that when the air is constantly removed from an enclosed system, the pressure linearly decreases with time. Although this vacuum-driven process was relatively weak (∼0.3% pressure loss after 30 s) compared to other approaches (e.g., vacuum pump), the mild process was suitable for the PANDA system without generating a high pulling force due to the sudden change of pressure. The result without using the holding layer indicated that the pressure gradually reached a plateau after 30 s of constant withdrawal of air. Simultaneously, we observed that most of the hanging drops failed to adhere to the bottom surface of the well plate due to the lack of the holding layer. The empty wells allowed more air to enter into the air chamber, balancing the pressure loss generated from the vacuum. Unlike the well plate without the holding layer, the pressure in the holding layer with U_0.25_/L_0.1_ design gradually decreased with time and reached to −220 Pa when all 49 hanging drops were formed. Interestingly, the fluctuation of the curve was observed after 15 s of constant withdrawal. To further investigate these fluctuations during the air withdrawal process, we fabricated a 9-well PANDA system and followed the same operation procedure for the formation of the hanging drops (Supplementary Figure S4). The internal pressure inside the 9-well air chamber was found to follow a similar trend as the 49-well chip, resulting in a reduction of the pressure with time. Furthermore, the change in the fluctuations was found to be more obvious than in the 49-well chip. A total of 9 peaks was observed from the pressure curve, indicating that the internal pressure slightly increased and decreased again due to the capillary force of the well during the formation process. These peaks were corresponding to the individual formation of the 9 hanging drops. The internal pressure eventually decreased to −220 Pa, demonstrating the same condition as the 49-well chip to form the last hanging drop. Therefore, the measurement of internal pressure in the PANDA system may become another tool to monitor the hanging drop formation process in real-time.

### Fabrication of 3D Kidney Microtissues Using PANDA Chip

**FIGURE 4:**
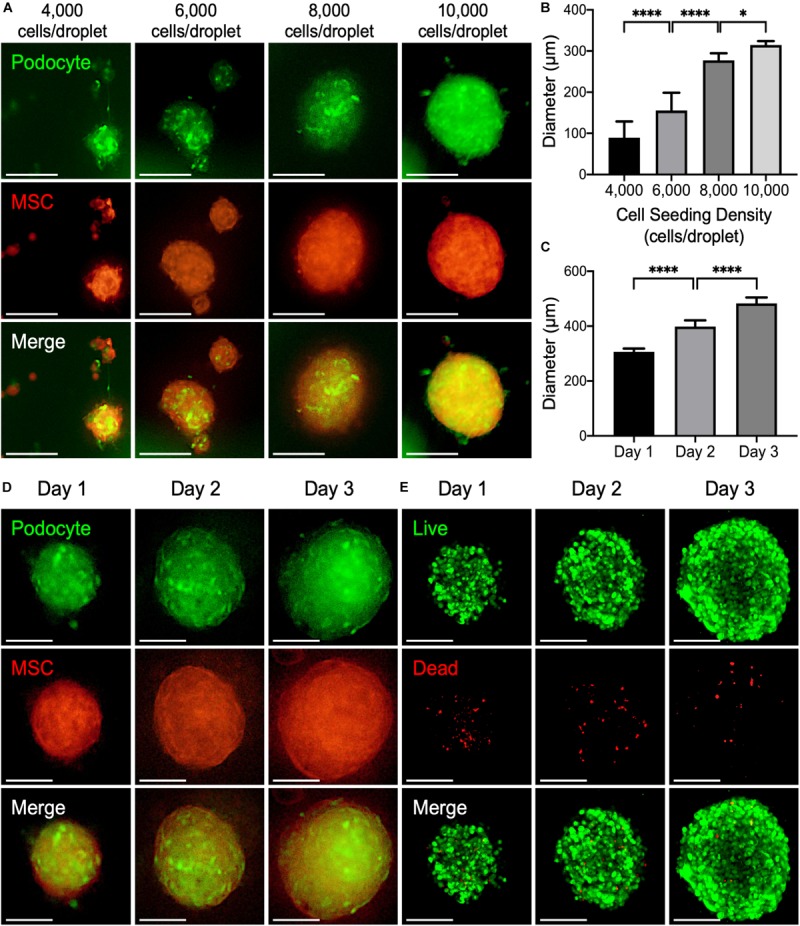
**(A)** Representative fluorescence images of 3D kidney microtissues comprised of podocyte (green) and mesenchymal stem cells (MSCs; red) fabricated using a PANDA chip. The microtissues were formed within 24 h. **(B)** The diameters of the formed 3D microtissues after 1 day of culture (*P < 0.05; ****P < 0.001; *N* = 20). **(C)** The diameters of the formed 3D microtissues cultured on different days (****P < 0.001. *N* = 20). **(D)** Representative fluorescence images of 3D kidney microtissues cultivated for various periods. **(E)** Confocal images of live (green) and dead (red) cells. All scale bars represent 200 μm.

To validate the application of the PANDA chip, 3D kidney microtissues that were composed of GFP-expressed podocytes and RFP-expressed MSCs in 1:1 ratio were formed. We first analyzed the effect of the cell number in each droplet on the formed microtissues. Within 24 h, cells within the droplet arrays assembled into 3D microtissues. According to the fluorescence images in Figure 4A, both GFP and RFP signals were detected from the obtained microtissues, demonstrating that the seeded podocytes and MSCs formed the 3D microtissues together.

Analysis of the assembled microtissues revealed a positive correlation between the number of cells in each droplet and the diameter of the 3D microtissues (Figure 4B). When the cell seeding density was relatively low (<6,000 cells per droplet), cells within one droplet tended to aggregate into multiple microtissues with various sizes and morphologies. Conversely, a higher cell seeding density (>8,000 cells per droplet) resulted in the formation of a single large microtissue in a droplet. Importantly, these microtissues exhibited a high consistency of diameters, which is critical for developing a drug screening platform. It is worth noting that in the group that contained 10,000 cells within each droplet, the podocytes within the assembled microtissues were distributed uniformly, suggesting enhanced interaction between podocytes and MSCs. Thereby, such a condition was chosen for fabricating 3D kidney microtissues for the following experiments.

During prolonged culture, an increase in the diameter of the microtissues up to ∼500 μm was observed after 3 days of culture (Figures 4C,D), suggesting that both podocytes and MSCs within the microtissues proliferated continuously. The similar result was also observed when only MSCs were cultured in the hanging drops for 3 days (Supplementary Figure S5). Owing to the diffusion limitation of oxygen (typically around 200–250 μm), a hypoxic core may develop within 3D multicellular microtissues, thus inducing cell death (Potapova et al., 2007; Kaully et al., 2009). To assess the viability of the prepared 3D microtissues, a live/dead assay that stains living cells with green fluorescence owing to the hydrolysis of calcein acetoxymethyl ester and dead cells with red fluorescence by ethidium homodimer was conducted. As indicated by the fluorescence images in Figure 4E, most cells grown within the 3D microtissues were alive during the entire culture period according to the prevalent green fluorescence emitted from the living cells.

**FIGURE 5:**
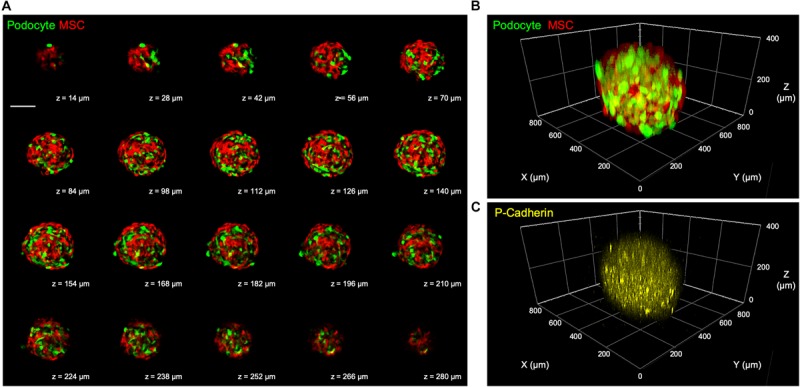
**(A)** Representative confocal Z-stack images showing mixed podocytes and MSCs in the fabricated 3D kidney microtissues after a 3-day culture. Scale bar represents 200 μm. **(B)** Reconstructed 3D confocal images showing the distribution of the two cell types and **(C)** the podocyte-specific P-cadherin.

After a 3-day culture, the 3D microtissues were processed for confocal microscopy. According to the obtained confocal Z-stack images, the harvested 3D kidney microtissues were composed of mixed podocytes and MSCs, as indicated by the signals of GFP and RFP, respectively, in each optical section (Figure 5A) and the 3D reconstructed image (Figure 5B and Supplementary Video S2). Additionally, podocyte-specific marker P-cadherin was identified throughout the whole 3D microtissues (Figure 5C), demonstrating the formation of kidney-like tissues.

To bridge the gap between *in vitro* cell-based assays and *in vivo* studies, a model that can appropriately reflect the complexities of the 3D microenvironment is highly warranted. It has been demonstrated that the cells grown within multicellular microtissues are exposed to a native-mimicking and heterogeneous microenvironment in terms of their interaction with neighboring cells and extracellular matrix, the acquisition of oxygen and nutrients, and the elimination of metabolic waste (Lin and Chang, 2008). The developed PANDA system can be employed to generate 3D microtissues with tunable cellular compositions and microenvironments, thus offering a platform that can engineer the desired artificial niches for modulating cell behaviors or elucidating cellular and molecular mechanisms. For example, by adjusting the density of cells seeded into a PANDA chip, the size of the formed microtissues and thus the internal heterogenous microenvironment with gradients of oxygen and nutrients can be established and precisely controlled, thereby providing a highly reproducible model for further analysis. Moreover, the successful incorporation of multiple cell types into a single microtissue demonstrates the potential to develop PANDA system for engineering cellular heterogeneity, an important characteristic of native tissue.

## Conclusion

In conclusion, we have demonstrated a new approach for fast and effective production of a hanging drop array with a consistent droplet volume using the PANDA system driven by withdrawing the air to generate pressure difference in the chip. An array of highly consistent 3D kidney microtissues were successfully fabricated using the PANDA system from composing kidney glomerular podocytes and mesenchymal stem cells, suggesting a potential application to serve as a rapid and economical platform to generate desired 3D microenvironments for exploring and harnessing cellular behaviors and responses. We envision that the PANDA system may be adopted to prepare various types of 3D microtissues, which can be employed as *in vivo*-mimicking models for a wide variety of biomedical research fields.

## Data Availability Statement

All datasets generated for this study are included in the article/Supplementary Material.

## Author Contributions

C-YC, T-HC, and J-HH contributed to the conception of the study. C-YC, T-HC, W-YY, and L-HH carried out the experiments. C-YC and W-YY helped perform the analysis with constructive discussions. C-CH and J-HH supervised the research design and wrote the manuscript. All authors approved the final version of the manuscript.

## Conflict of Interest

The authors declare that the research was conducted in the absence of any commercial or financial relationships that could be construed as a potential conflict of interest.
