# Supplementary material for: Development of a Novel Hanging Drop Platform for Engineering Controllable 3D Microenvironments
Source: Front Cell Dev Biol. 2020 May 7;8:327. doi: 10.3389/fcell.2020.00327 (PMC7221142; doi:10.3389/fcell.2020.00327)
Supplement: Supplementary file 1 [file Table_1.docx]

**Supplementary Material**

Development of a Novel Hanging Drop Platform for
Engineering Controllable 3D Microenvironments

Chin-Yi Cho^1#^, Tzu-Hsiang Chiang^1#^, Li-Hung Hsieh^2^, Wen-Yu Yang^2,3^, Hsiang-Hao Hsu^4,5^, Chih-Kuang Yeh^3^, Chieh-Cheng Huang^2^*, Jen-Huang Huang^1^*

^1^ Department of Chemical Engineering, National Tsing Hua University, Hsinchu, Taiwan

^2^ Institute of Biomedical Engineering, National Tsing Hua University, Hsinchu, Taiwan

^3^ Department of Biomedical Engineering and Environmental Sciences, National Tsing Hua University, Hsinchu, Taiwan

^4^ Kidney Research Center, Department of Nephrology, Linkou Chang Gung Memorial Hospital, Taoyuan, Taiwan

^5^ Department of Medicine, Chang Gung University, Taoyuan, Taiwan

Video S1: The top view of the hanging drop formation process from the 49-well PANDA chip.

Video S2: Reconstructed 3D confocal images showing the microtissue composed of podocytes (green) and MSCs (red). Scale bar represents 200 µm.

**
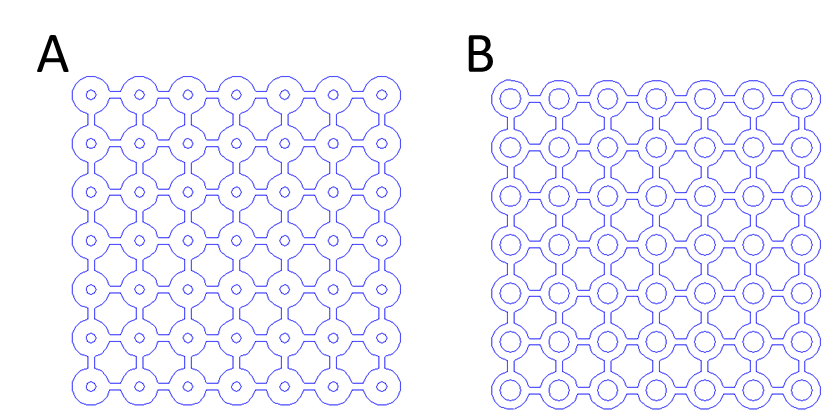
**

**Figure S1**. (A) The 2D design of the upper part of the holding layer. (B) The 2D design of the lower part of the holding layer. The upper part has 4.6 mm outer diameter and 1.2 mm inner diameter while the lower part has 4.6 mm outer diameter and 2.6 mm inner diameter.

**
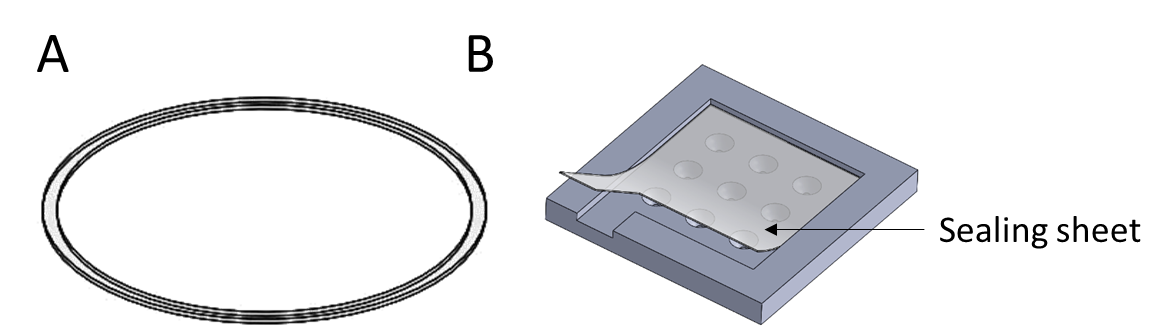
**

**Figure S2**. (A) The 2D design of the sealing ring. (B) The 2D design of the sealing sheet.

**
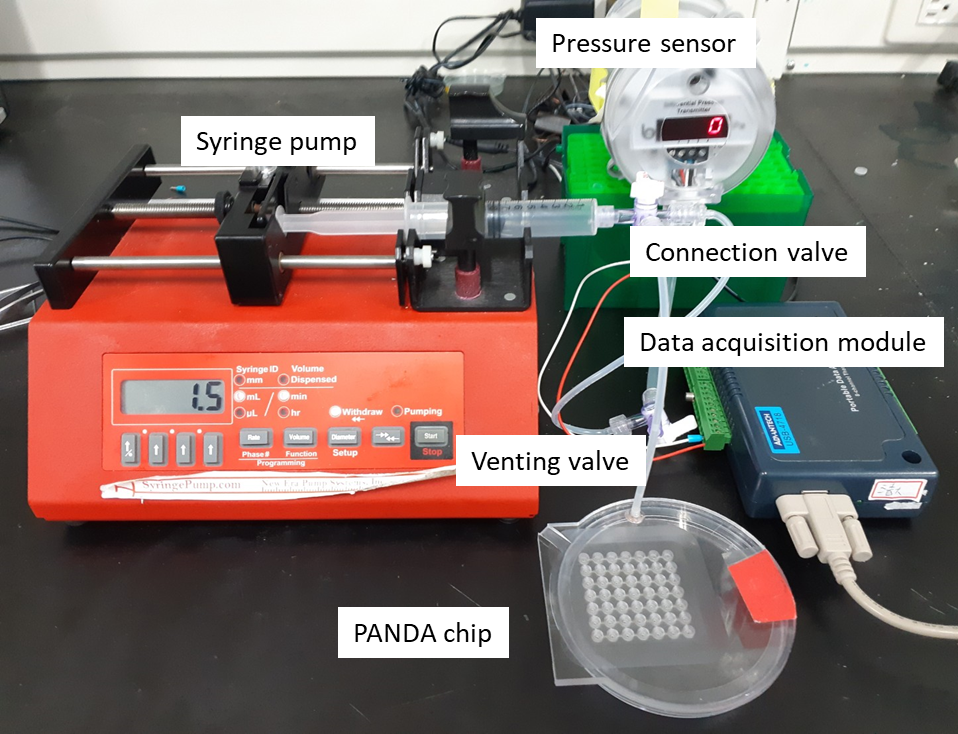
**

**Figure S3**. A pressure sensor system connected with the PANDA chip to measure the internal pressure inside the PANDA chip. The internal pressure is analyzed by the pressure sensor while the data is acquired by using the data acquisition module.

**
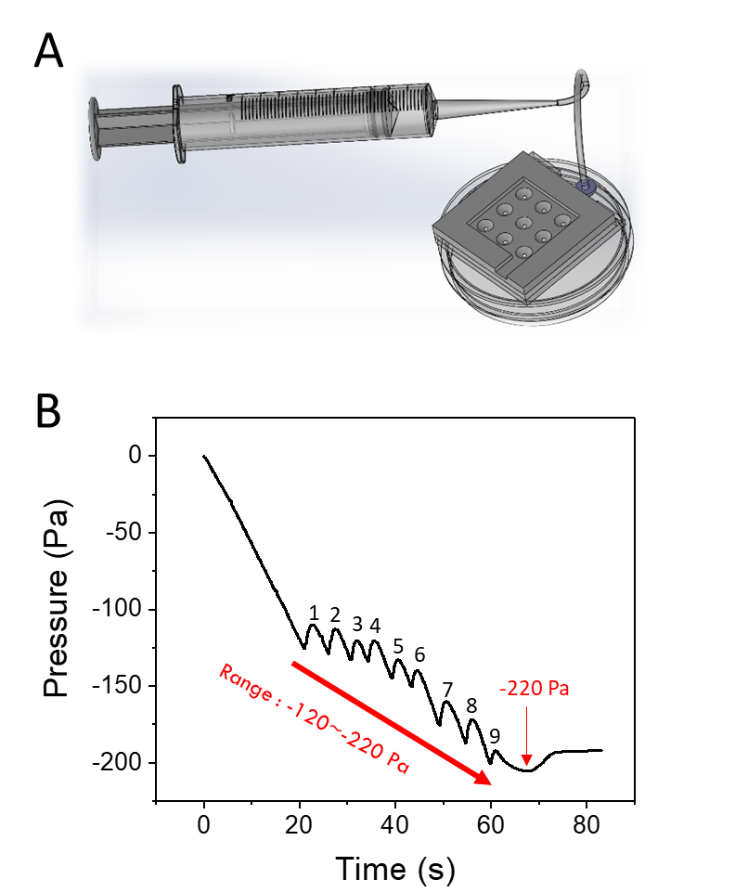
**

**Figure S4**. (A) The schematic of the 9-well PANDA chip connected to a syringe. (B) The internal pressure of the 9-well PANDA chip.


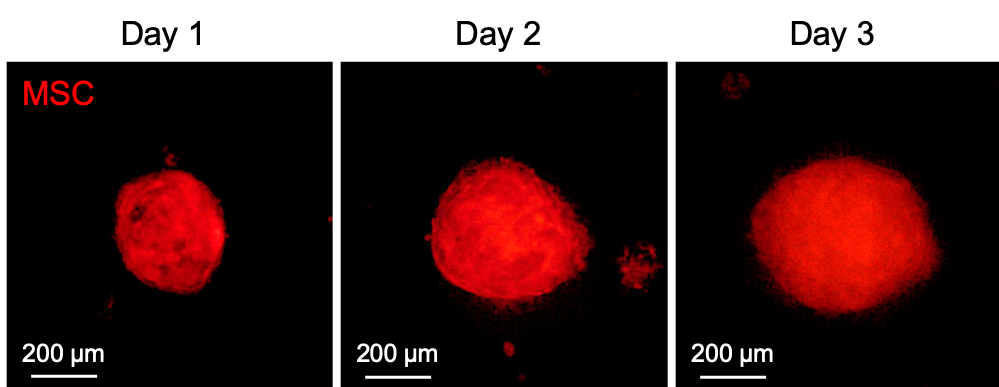


**Figure S5**. The red fluorescence images of MSC microtissue cultivated for 3 days. The diameter of MSC microtissue increased every day. Scale bars represent 200 µm.
